# Efficacy and Safety of Dupilumab in Moderate-to-Severe Bullous Pemphigoid

**DOI:** 10.3389/fimmu.2021.738907

**Published:** 2021-10-14

**Authors:** Yihua Zhang, Qiuyun Xu, Lihong Chen, Jiawen Chen, Jing Zhang, Ying Zou, Ting Gong, Chao Ji

**Affiliations:** ^1^ Department of Dermatology, The First Affiliated Hospital of Fujian Medical University, Fuzhou, China; ^2^ Central Laboratory, The First Affiliated Hospital of Fujian Medical University, Fuzhou, China

**Keywords:** bullous pemphigoid, dupilumab, IL-4/IL-13, pruritus, corticosteroid-spare

## Abstract

**Background:**

Bullous pemphigoid (BP) is an autoimmune blistering disorder that predominantly affects the elderly. As the main treatment for BP, systemic corticosteroids are often limited by their side effects. Safer treatment modalities are therefore needed. Dupilumab is a biologic agent used to treat BP in recent years.

**Methods:**

Medical records of patients with moderate-to-severe BP were retrospectively reviewed. Twenty-four patients were included (follow-up period: 32 weeks), eight of whom received dupilumab in combination with methylprednisolone and azathioprine (dupilumab group) while the other 16 patients received methylprednisolone and azathioprine (conventional group). Response to dupilumab was evaluated by comparison of several parameters (time to stop new blister formation, time to reduce the systemic glucocorticoids to minimal dose, and total amount of methylprednisolone).

**Results:**

The median age of patients in the dupilumab and conventional groups were 64.50 years (range: 22–90 years) and 64.50 years (range: 17–86 years), respectively. The median duration of disease before admission in the dupilumab group was 2 months (range: 1–240 months) and 2.5 months (range: 1–60 months) in the conventional group. The median time to stop new blister formation was 8 days (range: 1–13 days) and 12 days (range: 5–21 days) in patients of the dupilumab and conventional groups, respectively (*p* = 0.028 by Kaplan-Meier analysis). In addition, the median time to reduce the systemic glucocorticoids to minimal dose (methylprednisolone 0.08 mg/kg/day) was 121.5 and 148.5 days for the dupilumab and conventional therapy groups, respectively (*p* = 0.0053 by Kaplan-Meier analysis). The median total amount of methylprednisolone (at the time of reaching the minimal dose) used in the dupilumab group was 1,898 mg (range: 1,624–2,932 mg) while the cumulative dose of conventional group was 2,344 mg (range: 1,708–4,744 mg) (*p* = 0.036 by Mann-Whitney *U* test). The median total amount of azathioprine (at the time of reaching the minimal dose) used in dupilumab group was 8,300 mg (range: 7,100–10,400 mg) while the total dose of conventional group was 10,300 mg (range: 8,900–14,400 mg) (*p* = 0.0048 by Mann-Whitney *U* test). No adverse event related to dupilumab was recorded.

**Conclusions:**

Dupilumab in addition to methylprednisolone and azathioprine seems superior to methylprednisolone/azathioprine alone in controlling disease progression and accelerating the tapering of glucocorticoids.

## Introduction

Bullous pemphigoid (BP) is the most common autoimmune subepidermal blistering disease of the skin and primarily affects the elderly, especially those over the age of 70 years (1). The incidence of BP is increasing annually, with a global incidence of 2.4–21.7 individuals per million population (2–4). The pathogenesis is still unclear. BP180 and BP230 are two kinds of target antigens mainly involved in blister formation (5–7). In addition, type 2 proinflammatory cytokines, including interleukin-4 (IL-4) and interleukin-13 (IL-13), have been detected in blister fluid or skin biopsies of BP patients (8). Traditional therapies are limited by their side effects and their efficacy in preventing relapses of the disease (9). In recent years, biologic agents such as omalizumab (10–14) and rituximab (15–17) are widely used for refractory BP cases. Yet, there are very few reports suggesting the potential use of dupilumab in patients with BP (18–21). Dupilumab is a fully human IgG4 monoclonal antibody directed against the IL-4 receptor alpha (IL-4Rα) subunit that inhibits the signaling of IL-4 and IL-13, two type 2 cytokines (22). Herein, we conducted a retrospective study of dupilumab combined with methylprednisolone and azathioprine versus methylprednisolone and azathioprine for the treatment of patients with moderate-to-severe BP. 

## Methods

### Patients

Patients were eligible for inclusion if they exhibit:

a) Presence of tense bullae on examination and a clinical picture consistent with BP. (A clinical picture consistent with BP: multiple itchy erythema and urticaria on the skin; multiple tense bullae and erosion on the skin.)
b) Linear deposits of IgG and/or C3 at the dermoepidermal junction by direct immunofluorescence microscopy.
c) Binding of IgG along the epidermal side by indirect immunofluorescence microscopy on human salt-split skin or serum IgG reactivity against BP180 and/or BP230 by immunoblotting or enzyme-linked immunosorbent assay.
d) Moderate-to-severe BP: based on single BPDAI (perform evaluation of the following 1–3, and adopt the highest score) (23, 24)
  1. Skin: erosions/blisters total score of BPDAI: mild <15, moderate 15–34, severe >34;
  2. Skin: urticaria/erythema total score of BPDAI: mild <20, moderate 20–34, severe >34;
  3. Mucosa: erosions/blisters total score of BPDAI: mild <10, moderate 10–24, severe >24.

Because the skin lesions of patients in both groups were mainly blisters and/or erosions, the “Skin: erosions/blisters total score of BPDAI” was adopted to determine the severity in our study.

We reviewed 24 patients with moderate-to-severe BP to evaluate the effects of the IL-4/IL-13 antagonist dupilumab (follow-up periods: 32 weeks). Eight of them received dupilumab combined with methylprednisolone and azathioprine (dupilumab group), and the other 16 patients received methylprednisolone and azathioprine (conventional group). No randomization was performed. Patients before June, 2020 were included in the conventional group and patients after June, 2020 in the dupilumab group. Clinical and hematological examination data were analyzed to determine the treatment outcomes. There was no significant difference between the groups in the patients’ baseline data. Basic demographic information for the subjects is shown in **Table 1**.

**Table 1:** Baseline characteristics of the patients in both groups.

Characteristic	Dupilumab group	Conventional group	All patients
Number	8	16	24
Age (years; median (IQR))	64.50 (45.5–71.75)	64.50 (52.25–73.5)	64.50 (50.5–72.0)
Male	3	6	9
Female	5	10	15
Duration before admission(months; median (IQR))	2 (1.25–49.5)	2.5 (1.0–8.75)	2 (1.0–18.0)
IgE within the normal range			
Yes	4	7	11
No	4	9	13
EOS% within the normal range			
Yes	4	3	7
No	4	13	17
Underlying diseases			
Cardiovascular disease	3	2	5
Neurologic disorders	1	0	1
Hyperlipidemia	3	1	4
Diabetes	0	0	0
Cancers	2	1	3

### Treatment

**Figure 1:**
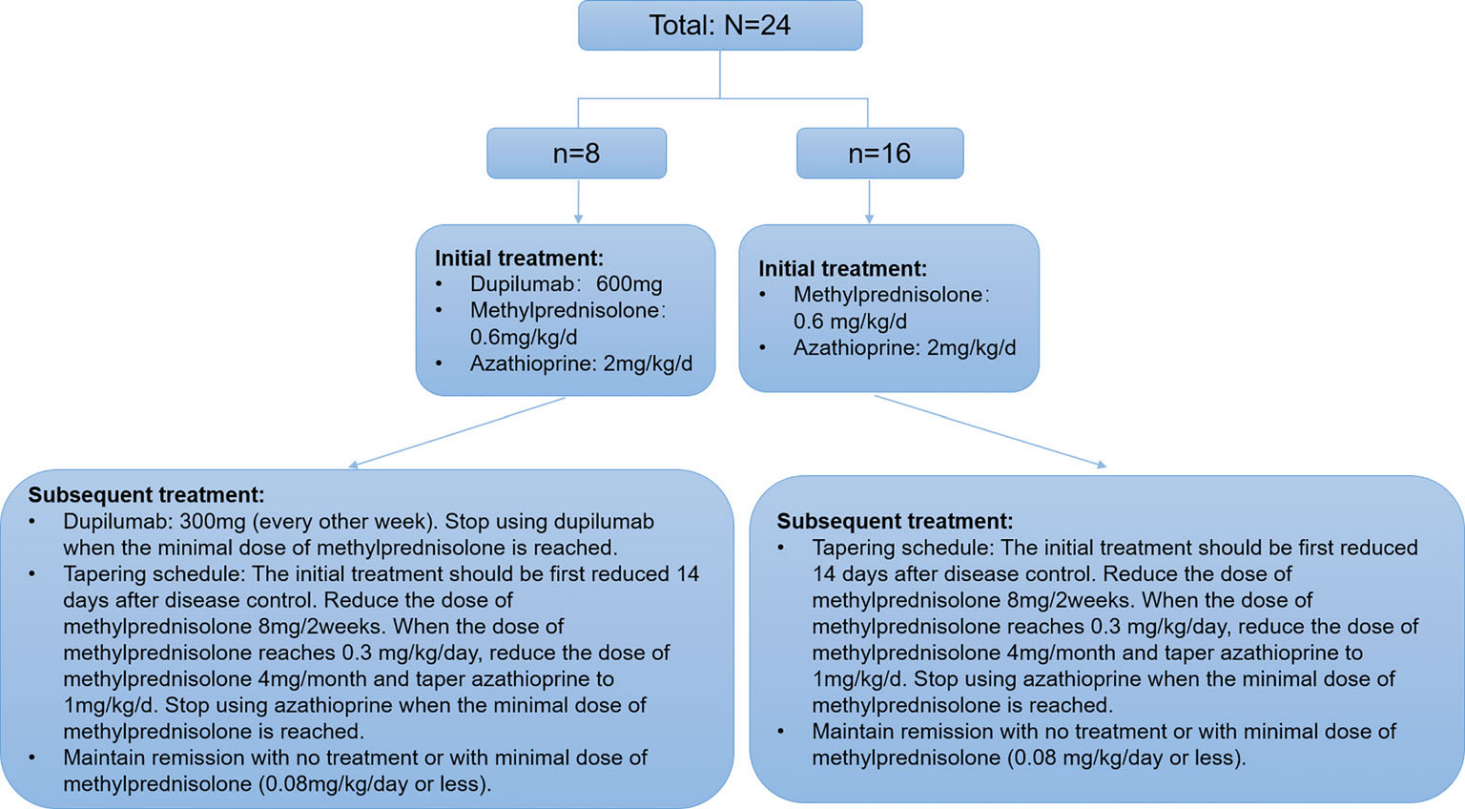
Algorithm describing the distribution, the treatments, and taper schedule received by the patients with moderate-to-severe BP.

Patients in the dupilumab group first received 600 mg dupilumab (induction dose) and then 300 mg every other week *via* subcutaneous injection. Concurrently, all participants in both groups received methylprednisolone (0.6 mg/kg/day) and azathioprine (2 mg/kg/day). All patients’ thiopurine s-methyltransferases were within normal levels. For both groups, the tapering schedule was identical where the initial treatment was first reduced 14 days after disease control (**Figure 1**). Disease control was defined as the point at which new lesions or pruritic symptoms ceased to form and established lesions began to heal (24). Minimal therapy was defined as <0.08 mg/kg/day of methylprednisolone and/or minimal adjuvant or maintenance therapy. Minimal adjuvant therapy and/or maintenance therapy was defined as following doses or less: azathioprine 0.7 mg/kg/day (with normal thiopurine s-methyltransferase level) (24). All subjects signed a consent form before treatment with dupilumab as currently required by Chinese authorities.

### Outcomes

Primary outcomes:

- Time to stop new blister formation.
- Time to reduce the systemic glucocorticoids to minimal dose (methylprednisolone 0.08 mg/kg/day).
- Hospitalization duration.

Secondary outcomes:

- The level of itch was measured with Numeric Rating Scale (NRS) (weeks 0, 1, and 2). NRS was graded from 0, no itch, to 10, insupportable itching.
- Bullous Pemphigoid Disease Area Index (BPDAI) activity score (week 0, week 2).
- Eosinophil counts (week 0, week 2) and IgE (week 0, week 2).
- Clinical remission (32 weeks): definition of clinical remission was adopted from Murrell et al. (24):
  - Complete remission off therapy: absence of new or established lesions or pruritus while patient is off all BP therapy for 2 months.
  - Complete remission on minimal therapy: absence of new or established lesions or pruritus while patient is receiving minimal therapy for at least 2 months.
  - Partial remission off therapy: presence of transient new lesions that heal within 1 week without treatment while patient is off all BP therapy for at least 2 months.
  - Partial remission on minimal therapy: presence of transient new lesions that heal within 1 week while patient is receiving minimal therapy for at least 2 months.
  - Mild new activity: <3 lesions/month (blisters, eczematous lesions, or urticarial plaques) that do not heal within 1 week or extension of established lesions or pruritus once/week but less than daily in patient who has achieved disease control; these lesions have to heal within 2 weeks.
  - Relapse/flare: appearance of >3 new lesions/month (blisters, eczematous lesions, or urticarial plaques) or at least one large (>10 cm diameter) eczematous lesion or urticarial plaques that do not heal within 1 week, or extension of established lesions or daily pruritus in patient who has achieved disease control.

### Statistical Analysis

In the present study, all data were analyzed using Kaplan-Meier method, two-way RM ANOVA test, and the Mann-Whitney *U* test (GraphPad Software). All data were presented as medians and interquartile ranges (IQRs) for descriptive purposes. *p*-Values were two-sided and performed with the appropriate statistical tests using GraphPad Prism software 8.0.1. A significant difference was considered to be present at *p* < 0.05.

## Results

We report the results of primary outcomes and selected secondary outcomes. The median age of patients in the dupilumab group was 64.50 years (range: 22–90 years) and 64.5 years (range: 17–86 years) of patients in the conventional group. The median duration of disease before admission in patients in the dupilumab group was 2 months (range: 1–240 months) and 2.5 months (range: 1–60 months) in patients in the conventional group, respectively. The median duration of dupilumab treatment in patients in the dupilumab group was 4 months (range: 3–6 months). There was no significant difference in the baseline data of these patients (**Table 1**).

### Primary Outcomes in the Dupilumab Group Compared With the Conventional Group

**Figure 2:**
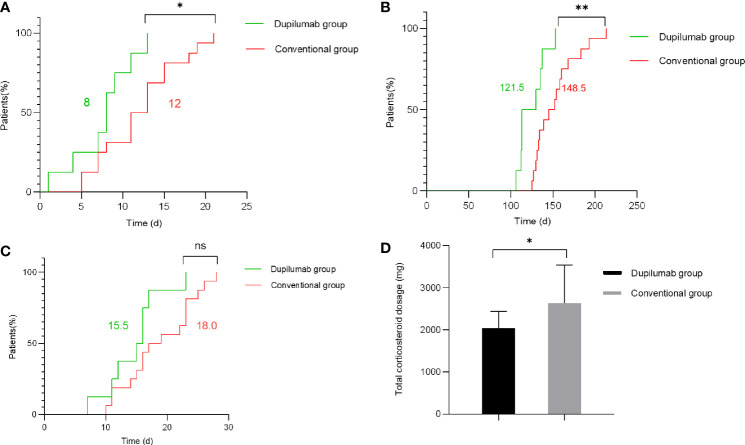
Primary outcomes in the dupilumab group compared with the conventional group. **(A)** Time to stop new blister formation (*p* = 0.028 by Kaplan-Meier analysis). **(B)** Time to reduce the systemic glucocorticoids to minimal dose (methylprednisolone 0.08 mg/kg/day) (*p* = 0.0053 by Kaplan-Meier analysis). **(C)** Hospitalization duration (*p* > 0.05 by Kaplan-Meier analysis). The numbers in green and red represent the median number of days for the dupilumab group and conventional group, respectively. **(D)** The total amount of methylprednisolone administered to patients in the dupilumab group and conventional group (at the time of reaching the minimal dose) (*p* = 0.036 by Mann-Whitney *U* test) (^*^ *p* < 0.05; ^**^ *p* < 0.01; ns, not significant).

The primary parameters included time to stop new blister formation, time to reduce the systemic glucocorticoids to minimal dose (methylprednisolone 0.08 mg/kg/day), and hospitalization duration. Compared with the conventional group, we found that the dupilumab group was associated with shorter time to stop new blister formation (**Figure 2A**) in patients with BP (*p* = 0.028 by Kaplan-Meier analysis). Specifically, the median time to cessation of new blisters was 8.0 and 12.0 days in the dupilumab and the conventional groups, respectively. Additionally, time to reduce the systemic glucocorticoids to minimal dose (**Figure 2B**) was significantly shorter in patients with dupilumab treatment (*p* = 0.0053 by Kaplan-Meier analysis). For patients in the dupilumab and conventional groups, the median time to reduce the systemic glucocorticoids to minimal therapy were 121.5 and 148.5 days, respectively. The median total amount of methylprednisolone (at the time of reaching the minimal dose) used in dupilumab group was 1,898 mg (range: 1,624–2,932 mg) while the cumulative dose of conventional group was 2,344 mg (range: 1,708–4,744 mg) (**Figure 2D**; *p* = 0.036 by Mann-Whitney *U* test). The median total amount of azathioprine (at the time of reaching the minimal dose) used in the dupilumab group was 8,300 mg (range: 7,100–10,400 mg) while the total dose of the conventional group was 10,300 mg (range: 8,900–14,400 mg) (**Supplementary Figure 2D**; *p* = 0.0048 by Mann-Whitney *U* test). To our surprise, there was no significant difference in hospital duration between the groups (**Figure 2C**). However, our relatively small sample size may limit what differences can be observed.

### Assessment of Secondary Outcomes in Both Groups

**Figure 3:**
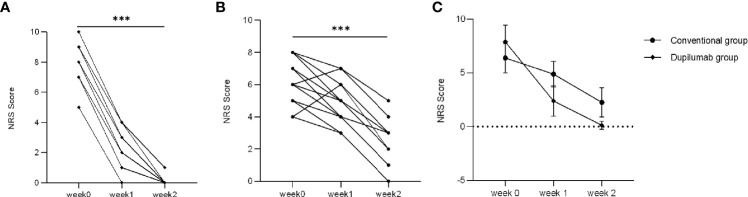
Comparison of NRS score between two treatment groups. NRS score has decreased to varying degrees from weeks 0 to 2 in the dupilumab group **(A)** (^***^ *p* < 0.001 by Mann-Whitney *U* test), and the conventional group **(B)** (^***^ *p* < 0.001 by Mann-Whitney *U* test). **(C)** Patients in dupilumab group showed more privileges in relieving itch (*p* = 0.034 by two-way RM ANOVA).

Secondary parameters included NRS score, BPDAI activity score, and counts of eosinophil and IgE. NRS score was assessed at different time points (weeks 0, 1, and 2). Patients had a significant itch with an NRS score ranging from 4 to 10 at week 0 in both groups. The NRS score had decreased to varying degrees in both groups at week 2 (**Figure 3A**: dupilumab group; **Figure 3B**: conventional group; *p* < 0.001 by Mann-Whitney *U* test). In our study, patients in the dupilumab group showed more privileges in relieving itch (**Figure 3C**, *p* = 0.034, two-way RM ANOVA). However, there was no significant difference between the two groups in NRS score from weeks 2 to 32 (**Supplementary Figure 2A**; *p* > 0.05, two-way RM ANOVA).

**Figure 4:**
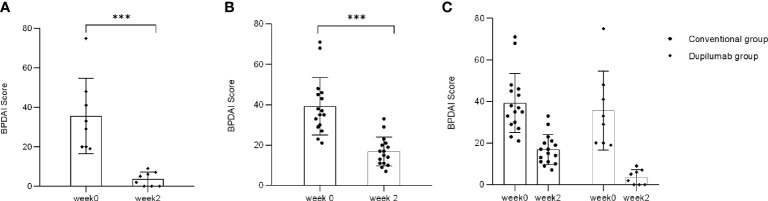
Comparison of single BPDAI (skin: erosions/blisters) score between two treatment groups. BPDAI score has declined to varying degrees from weeks 0 to 2 in the dupilumab group **(A)** (^***^ *p* < 0.001 by Mann-Whitney *U* test) and the conventional group **(B)** (^***^ *p* < 0.001 by Mann-Whitney *U* test). **(C)** BPDAI score decreased more rapidly in patients in the dupilumab group than the conventional group (*p*= 0.0308 by two-way RM ANOVA).

BPDAI activity score was used as an international standard to evaluate disease severity of BP. There was the single BPDAI (skin: erosions/blisters) score used in severity determination. The median BPDAI activity score in patients in the dupilumab group was 34.25 (range: 19–75) at week 0 and 3.7 (range: 0–9) at week 2, while that of the conventional group was 36 (range: 21–71) and 16 (range: 7–33), respectively. BPDAI activity score significantly decreased from weeks 0 to 2 in both groups (**Figure 4A**: dupilumab group, **Figure 4B**: conventional group; *p* < 0.001 by Mann-Whitney *U* test). Consistent with the NRS score data, it has been indicated that patients’ BPDAI activity score declined more rapidly in the dupilumab group (**Figure 4C**, *p* = 0.0308, two-way RM ANOVA). However, there was no significant difference between the two groups in BPDAI score from weeks 2 to 32 (**Supplementary Figure 2B**; *p* > 0.05, two-way RM ANOVA).

**Figure 5:**
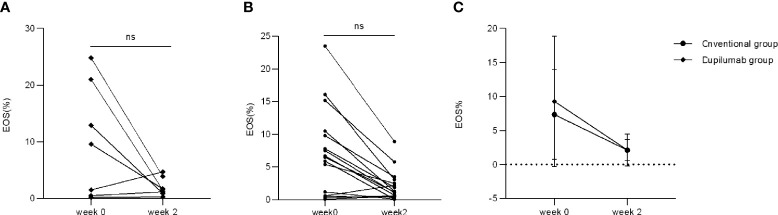
Comparison of EOS% between two treatment groups. EOS% has decreased to varying degrees from weeks 0 to 2 in the dupilumab group **(A)** (ns, not significance; *p* > 0.05 by Mann-Whitney *U* test) and the conventional group **(B)** (ns, not significance; *p* > 0.05 by Mann-Whitney *U* test). **(C)** Comparison of improvement in EOS% in both groups (*p* > 0.05 by two-way RM ANOVA).

Only four patients in the dupilumab group had an increased percentage of eosinophils (EOS%) at week 0 (range: 9.6%–24.8%) while the other four patients’ EOS% were within the normal range. There were 11 patients in the conventional group with increased EOS% at week 0 (range: 5.4%–23.5%) while the other five patients’ EOS% were within the normal range. All patients showed normal EOS% at week 2 in the dupilumab group (**Figure 5A**; *p* > 0.05, Mann Whitney *U* test) while two patients retained an increased EOS% in the conventional group (**Figure 5B**; *p* > 0.05, Mann Whitney *U* test). There was no significant difference in decreasing patients’ EOS% between the dupilumab and conventional groups (**Figure 5C** *p* > 0.05, two-way RM ANOVA).

**Figure 6:**
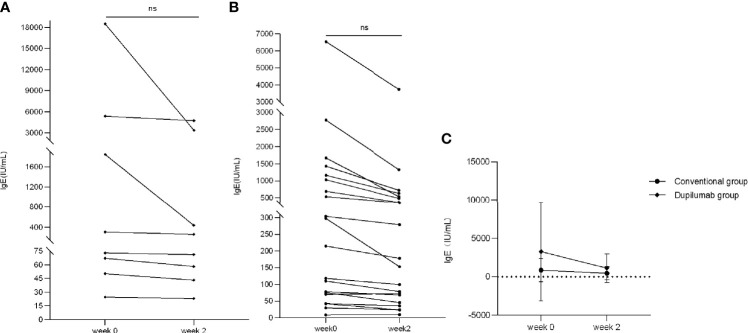
Comparison of IgE between two treatment groups. IgE has decreased to varying degrees from weeks 0 to 2 in the dupilumab group **(A)** (ns, not significant; *p* > 0.05 by Mann-Whitney *U* test) and the conventional group **(B)** (ns, not significant *p* > 0.05 by Mann-Whitney *U* test). **(C)** Comparison of improvement in IgE in both groups (*p* > 0.05 by two-way RM ANOVA).

In the dupilumab group, four patients had elevated counts of IgE (range: 308–18,500 IU/ml) while the other four patients were in the normal range at week 0. In the conventional group, 11 patients had elevated counts of IgE (range: 215–6,550 IU/ml) while the other five patients were in the normal range at week 0. We found that IgE counts decreased to varying degrees at week 2 in both groups with no significant statistical difference (**Figure 6A**: dupilumab group, **Figure 6B**: conventional group; *p* > 0.05, Mann-Whitney *U* test). In addition, there was no significant difference in declining patients’ IgE between groups (**Figure 6C**, *p* > 0.05, two-way RM ANOVA).

Clinical remission was another secondary outcome. The rates of patients who achieved complete remission (off therapy or with minimal therapy) were 62.5% and 56% in the dupilumab and conventional groups, respectively. Moreover, one patient (12.5%) in the dupilumab group and four patients (25%) in the conventional group obtained partial remission (off therapy or with minimal therapy). Three patients (18.75%) in the conventional group relapsed within 32 weeks while there was only one recurrence (12.5%) in the dupilumab group. For the four relapse cases during the dose-reduction period, the dose of methylprednisolone was increased to the previous level and topical corticosteroid was added (**Table 2**). There were no treatment failures in either group.

**Table 2:** Details of relapsed cases in both groups.

Number of patients	Age	Gender	Duration of disease	Group	Time to relapse	Systemic glucocorticoids dosage	BPDAI score (skin: erosions/blisters total score of BPDAI)	NRS score	Treatment options	Response to increased therapy
Patient 1	61	F	60 months	Dupilumab group	Week 13	4 mg/day	5 (skin: erosions/blisters)	3	Increased systemic glucocorticoids dosage to 8 mg/day and added TCS to the regimen	Improved within 1 week
Patient 2	86	M	6 months	Conventional group	Week 24	12 mg/day	10 (skin: erosions/blisters)	2	Increased systemic glucocorticoids dosage to 16 mg/day and added TCS to the regimen	Improved within 1 week
Patient 3	52	F	2 months	Conventional group	Week 20	8 mg/day	11 (skin: erosions/blisters)	4	Increased systemic glucocorticoids dosage to 12 mg/day and added TCS to the regimen	Improved within 2 weeks
Patient 4	56	M	48 months	Conventional group	Week 29	4 mg/day	8 (skin: erosions/blisters)	1	Increased systemic glucocorticoids dosage to 8 mg/day and added TCS to the regimen	Improved within 1 week

## Discussion

BP is an autoimmune blistering disease that is characterized by tense bullae and pruritus. The mortality rate of patients suffering from BP is as high as 6% to 41% (25–27). The conventional treatment with prolonged high-dose systemic corticosteroids is likely to cause unavoidable side effects. In BP, auto-antibodies directed against hemidesmosome proteins BP180 and/or BP230 lead to the development of subepidermal blisters (28). In addition, many studies have shown that type 2 cytokines, including IL-4 and IL-13, are involved in the pathogenesis of BP (29, 30).

Dupilumab is a human monoclonal antibody directed to IL-4Rα, which modulates type 2 inflammation by inhibiting IL-4 and IL-13 signaling (31). Yet, only a few reports have demonstrated that refractory BP can be potentially treated with dupilumab (18–21). Our study is a retrospective study to evaluate the efficacy and safety of dupilumab in combination with methylprednisolone and azathioprine in patients with moderate-to-severe BP compared with treatment with methylprednisolone and azathioprine alone.

We found that dupilumab showed certain superiority in the treatment of moderate-to-severe BP. It significantly reduced the time to stop new blister formation (8.0 vs. 12.0 days in the dupilumab and conventional groups, respectively, *p* = 0.028). On the other side, prolonged use of methylprednisolone may cause many side effects affecting multiple systems (32). In our study, dupilumab appeared to have a corticosteroid-sparing effect when duration of corticosteroid use in both groups was compared (121.5 vs. 148.5 days in the dupilumab and conventional therapy groups, respectively, *p* = 0.0053). The total amount of methylprednisolone used in the dupilumab and conventional groups also showed significant difference (1,898 vs. 2,344 mg in the dupilumab and conventional therapy groups, respectively, *p* = 0.036). In addition, the total amount of azathioprine used in the dupilumab and conventional groups also showed significant difference (8,300 vs 10,300 mg in the dupilumab and conventional therapy groups, respectively, *p* = 0.0048). It is obvious that the encouraging results are due to the early reduction of glucocorticoids and early progression to minimal therapy in the dupilumab group.

The baseline NRS scores of patients in both groups were ≥4. Our data showed that dupilumab significantly improved pruritus at week 2. This is consistent with the treatment of atopic dermatitis (AD) and significantly improves life quality (29, 31). Nevertheless, to our surprise, dupilumab showed no effect in accelerating the downregulation of the elevated eosinophil counts in BP patients in the current study. We think that this is because the systemic application of glucocorticoids alone is sufficient to reduce the level of peripheral blood eosinophils within a few hours (33). The efficacy of dupilumab in relieving pruritus may emphasize the type 2 immunity, which contributes to pruritus in AD and BP (19).

In the current study, patients in the dupilumab group showed higher complete remission rate (62.5% vs. 56% in the dupilumab and conventional groups, respectively). In addition, our study indicated that patients in the dupilumab group had a decreased rate of relapse than in the conventional group (12.5% vs. 18.75%). However, there was no significant difference between the two groups in clinical remission. We considered that this was because of the small sample size and short follow-up period. The ongoing enrollment will enlarge the sample size with longer follow-up to further verify the efficacy of dupilumab in treatment of patients with BP.

The adverse events in both groups were described in detail in **Table 3**. There was no serious adverse event observed in either group. Furthermore, it is worth mentioning that no adverse events related to dupilumab were found in our study.

**Table 3:** Details of adverse events in both groups.

Group	Dupilumab group	Conventional group
Adverse events	*N* = 8, *n* (%), severity	*N* = 16, *n* (%), severity
Osteoporosis^a^	1 (12.5), grade 1	2 (12.5), grade 1
Leukocytosis^b^	0	1/16 (6.25), grade 3
Total	1 (12.5)	4 (18.5)

a A disorder characterized by reduced bone mass, with a decrease in cortical thickness and in the number and size of the trabeculae of cancellous bone (but normal chemical composition), resulting in increased fracture incidence. Grade 1: radiologic evidence of osteoporosis or bone mineral density (BMD) t-score −1 to −2.5 (osteopenia). (CTCAE v5.0—27 November 2017).

b A disorder characterized by laboratory test results that indicate an increased number of white blood cells in the blood. Grade 3: >100,000/mm^3^. (CTCAE v5.0—27 November 2017).

**Figure 7:**
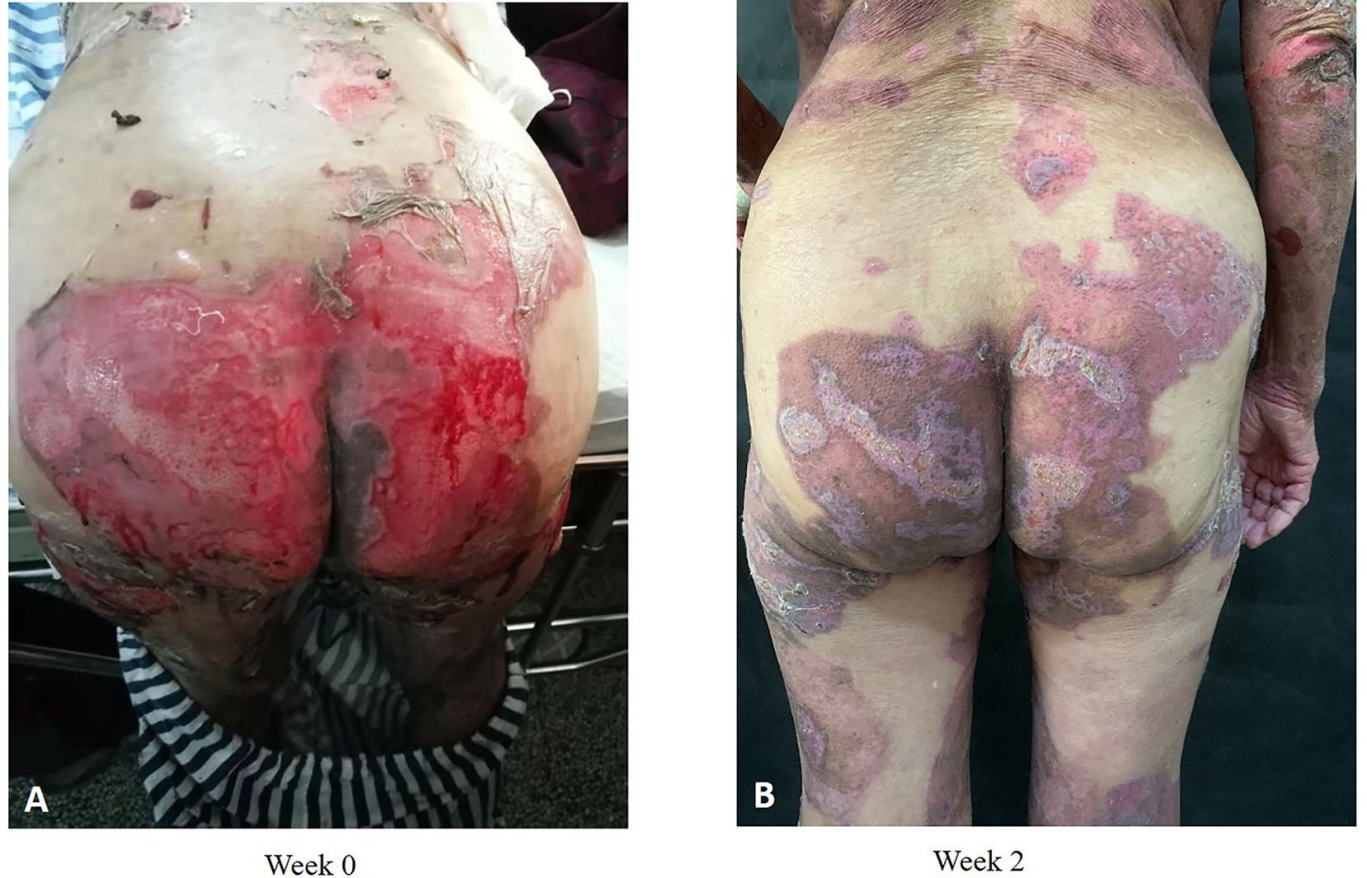
Clinical photographs of one patient with BP who received dupilumab in combination with conventional therapy. Admission **(A)**; 2 weeks after dupilumab (600 mg) treatment **(B)**.

Collectively, compared with other studies (18, 20, 34), our study determined the same advantage of dupilumab in BP treatment. The novelty that our study proposes for the first time is that dupilumab in addition to methylprednisolone and azathioprine seems superior to methylprednisolone/azathioprine alone in controlling disease progression and accelerating the tapering of glucocorticoids (**Figure 7**).

Potential limitations include the retrospective nature of the data, single-center nature, and small sample size. Our study highlights the effectiveness of dupilumab in BP treatment and hence deserves replication in larger samples and future randomized controlled trials. The ongoing study will allow for further analyses involving larger sample size with longer follow-up. 

## Conclusion

As a retrospective study to evaluate the efficacy and safety of dupilumab in patients with moderate-to-severe BP, dupilumab is found effective in combination with methylprednisolone and azathioprine compared with methylprednisolone and azathioprine alone. In addition, dupilumab showed a potential corticosteroid-sparing effect without significant side effects. This study provides useful guidance on the clinical use of dupilumab in the treatment of patients with moderate-to-severe BP. More studies are needed to confirm the efficacy and safety of dupilumab treatment. 

## Data Availability Statement

The original contributions presented in the study are included in the article/**Supplementary Material**. Further inquiries can be directed to the corresponding authors. 

## Ethics Statement

The studies involving human participants were reviewed and approved by the Medical Technology Clinical Application Ethics Committee of the First Affiliated Hospital of Fujian. Written informed consent for participation was not required for this study in accordance with the national legislation and the institutional requirements. Written informed consent was obtained from the individual(s) for the publication of any potentially identifiable images or data included in this article.

## Author Contributions

YHZ, QX, LC and CJ donceptualized and designed the study; YHZ, QX and LC wrote the manuscript; TG and CJ revised the article critically for important intellectual content. YHZ, YZ, JWC, JZ and QX collected clinical pictures and analyzed data. All authors contributed to the article and approved the submitted version.

## Conflict of Interest

The authors declare that the research was conducted in the absence of any commercial or financial relationships that could be construed as a potential conflict of interest.

## Publisher’s Note

All claims expressed in this article are solely those of the authors and do not necessarily represent those of their affiliated organizations, or those of the publisher, the editors and the reviewers. Any product that may be evaluated in this article, or claim that may be made by its manufacturer, is not guaranteed or endorsed by the publisher.
