# Supplementary material for: Efficacy and Safety of Dupilumab in Moderate-to-Severe Bullous Pemphigoid
Source: Front Immunol. 2021 Oct 14;12:738907. doi: 10.3389/fimmu.2021.738907 (PMC8552038; doi:10.3389/fimmu.2021.738907)
Supplement: Supplementary file 1 [file DataSheet_1.docx]

Supplementary Material

## Supplementary Figures


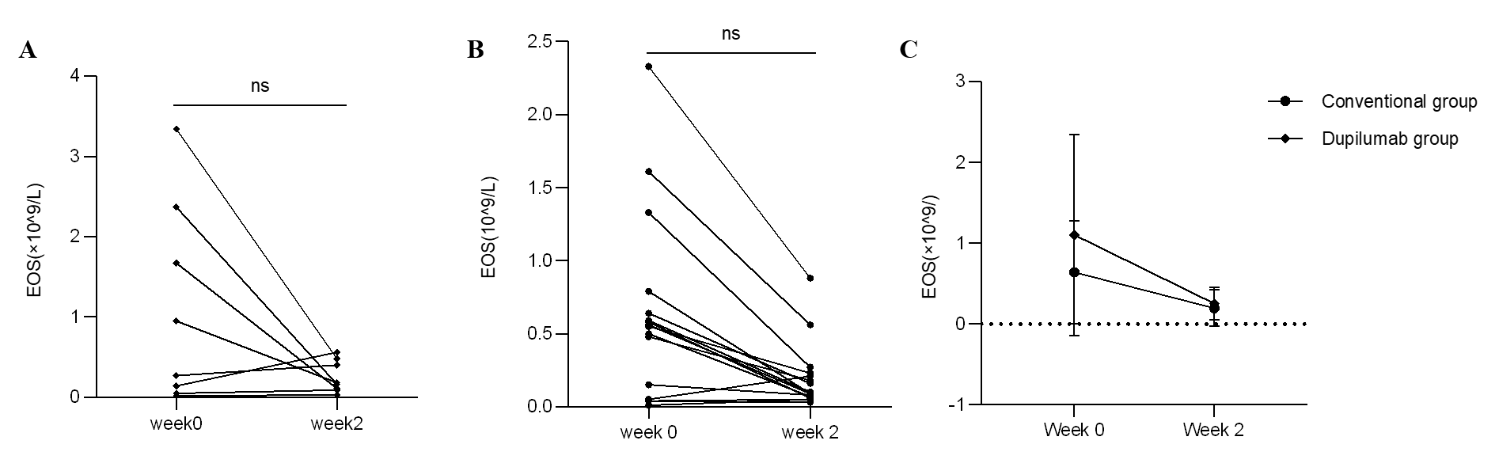


**Supplementary Figure 1.** Comparison of EOS counts between two treatment groups. EOS counts have decreased to varying degree from week 0 to week 2 in dupilumab group (A) (ns: none significance, *P*>0.05, by Mann-Whitney test), and the conventional group (B) (ns: none significance, *P*>0.05, by Mann-Whitney test). (C) Comparison of improvement in EOS counts in both groups (*P*>0.05, by two-way RM ANOVA).


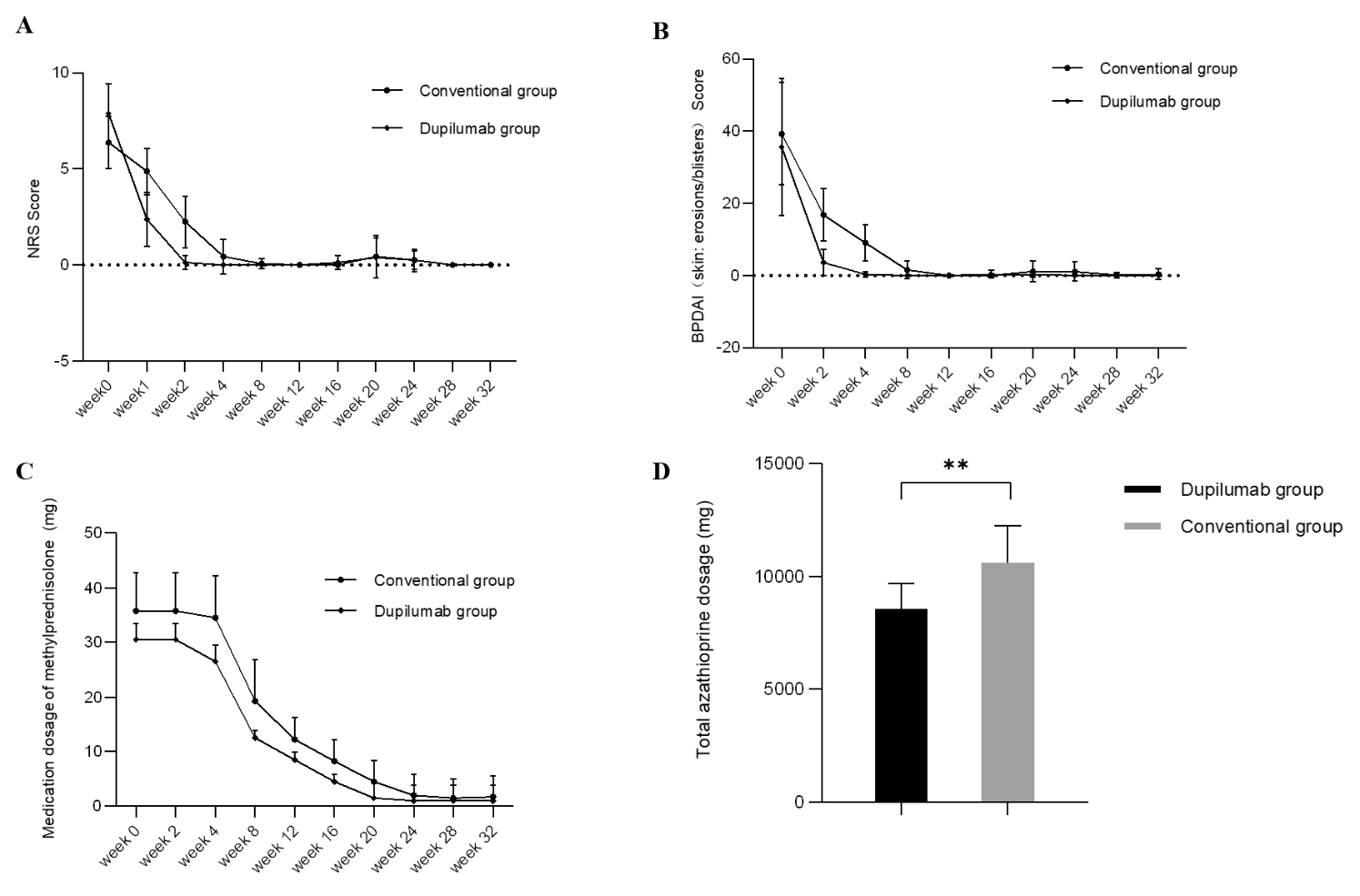


**Supplementary Figure 2.** The NRS score (A), BPDAI (skin: erosions/blisters) score (B), and the medication dosage of methylprednisolone (C) for both groups at various time points between the week 2 and week 32. (D) The total amounts of azathioprine used in dupilumab group and the conventional group also showed significant difference (8300 mg vs 10300 mg in dupilumab group and conventional therapy group, respectively, *P*=0.0048). All patients in two groups were followed up to 32 weeks.
